# High-resolution cryo-EM using beam-image shift at 200 keV

**DOI:** 10.1107/S2052252520013482

**Published:** 2020-10-29

**Authors:** Jennifer N. Cash, Sarah Kearns, Yilai Li, Michael A. Cianfrocco

**Affiliations:** a Life Sciences Institute, Department of Biological Chemistry, University of Michigan, Ann Arbor, MI 48109, USA

**Keywords:** single-particle cryo-EM, *RELION*, aldolase

## Abstract

Recent advances in single-particle cryo-electron microscopy (cryo-EM) data collection utilize beam-image shift to improve throughput. Despite implementation on 300 keV cryo-EM instruments, it remains unknown how well beam-image-shift data collection affects data quality on 200 keV instruments and the extent to which aberrations can be computationally corrected. To test this, a cryo-EM data set for aldolase was collected at 200 keV using beam-image shift and analyzed. This analysis shows that the instrument beam tilt and particle motion initially limited the resolution to 4.9 Å. After particle polishing and iterative rounds of aberration correction in *RELION*, a 2.8 Å resolution structure could be obtained. This analysis demonstrates that software correction of microscope aberrations can provide a significant improvement in resolution at 200 keV.

## Abstract 2

In this work, it is shown that significant microscope aberrations caused by beam-image-shift cryo-EM at 200 keV can be overcome using computational correction, improving a structure of aldolase from 4.9 to 2.8 Å resolution.

## 1. Introduction   

In order to increase the throughput from cryo-EM instruments, many laboratories and facilities have begun to use beam-image shift for data collection (Cheng *et al.*, 2018 ▸). Using this approach, instead of moving the stage to each position on the cryo-EM grid, a process that requires precise movement, the beam is moved in conjunction with image adjustments. Without long waiting times in moving the stage, tilting the beam leads to a dramatic increase in the number of exposures per hour. As such, it is now routine to use beam tilt to collect 100–300 exposures per hour, whereas previously it was only possible to collect 40–50 exposures per hour. This throughput will continue to increase with the advent of direct detectors with faster frame rates, leading to hundreds of exposures per hour.

Even though users can collect two to three times the amount of data using beam-image shift, they must overcome an additional aberration induced by the beam-image shift: beam tilt (Glaeser *et al.*, 2011 ▸). When using beam-image shift to collect exposures, the resulting image will have both axial and off-axis beam tilt (or coma), aberrations that will dampen the high-resolution (<3 Å) information in the micrographs (Glaeser *et al.*, 2011 ▸). Owing to this, it is common practice to minimize beam tilt in the cryo-EM instrument through microscope alignments ahead of data collection.

Axial beam-tilt aberrations can be corrected computationally for high-resolution structures. For example, this was implemented by Henderson and coworkers for the atomic resolution structure of bacteriorhodopsin from 2D crystals (Henderson *et al.*, 1986 ▸). Since its use 40 years ago, recent advances in single-particle cryo-EM have led to the incorporation of axial beam-tilt correction into software packages such as *RELION* (Zivanov *et al.*, 2018 ▸, 2020 ▸). The availability of axial beam-tilt correction has led to its widespread adoption in cryo-EM structure determination. Users are typically finding a 0.2–0.8 mrad beam tilt on previously aligned 300 kV Titan Krios instruments, and correction for this has led to modest improvements in resolution (typically 0.1–0.3 Å) (Herzik *et al.*, 2017 ▸; Wu *et al.*, 2020 ▸).

Even though beam-image-shift data collection in combination with aberration correction has been implemented for data sets at 300 keV, there is limited experimental data on the correction for beam tilt for data sets collected at 200 keV. Beam-image shift would likely induce coma, thus introducing optical aberrations, and we wanted to determine whether it can be overcome computationally. Given that the phase error caused by either axial or off-axis beam tilt scales with the wavelength (λ) squared (Glaeser *et al.*, 2011 ▸), changing from 300 keV (λ = 1.96 pm) to 200 keV (λ = 2.51 pm) will result in worse phase error from both axial and off-axis beam tilt. While previous work indicated that short-range beam-image shift could achieve a resolution of 3.3 Å for the T20S proteasome at 200 keV (Herzik *et al.*, 2017 ▸), this same work required the use of stage positioning to obtain a resolution better than 3 Å. As recently shown using these original data sets for aldolase and the T20S proteasome, *RELION*-3.1 now allows higher-order aberrations to be corrected computationally (Zivanov *et al.*, 2020 ▸). This allowed the resolution of aldolase to be improved from 2.5 to 2.1 Å and that of the T20S proteasome to be improved from 3.1 to 2.3 Å.

In order to test the limits of computational correction of microscope aberrations at 200 keV, we collected and analyzed a data set for aldolase using beam-image shift on a Talos Arctica at 200 kV. Using this data set, we were able to determine a 4.9 Å resolution structure of aldolase without aberration corrections. Following iterative rounds of axial beam-tilt correction and particle polishing, we were able to determine a 2.8 Å resolution structure of aldolase. This indicates that beam-image shift can be an effective data-collection strategy to increase the throughput on 200 keV cryo-EM instruments, where microscope aberrations can be corrected computationally.

## 2. Methods   

### 2.1. Sample preparation   

Pure aldolase isolated from rabbit muscle was purchased as a lyophilized powder (Sigma–Aldrich) and solubilized in 20 m*M* HEPES pH 7.5, 50 m*M* NaCl at 1.6 mg ml^−1^. The sample was dispensed onto freshly plasma-cleaned UltrAuFoil R1.2/1.3 300-mesh grids (Electron Microscopy Services) and applied to a grid in the chamber of a Vitrobot (Thermo Fisher) at ∼95% relative humidity and 4°C. The sample was blotted for 4 s with Whatman No. 1 filter paper immediately prior to plunge-freezing in liquid ethane cooled by liquid nitrogen.

### 2.2. Cryo-EM data acquisition and image processing   

The proper eucentric height of the specimen was determined using *Leginon* immediately before starting data collection. Parallel illumination of the beam was achieved earlier in the previous week by first adjusting the defocus to bring the objective aperture into focus in the front focal plane of the diffraction lens in diffraction mode followed by adjustments of the beam intensity to minimize the spread of diffraction. Data were acquired using the *Leginon* automated data-acquisition program (Suloway *et al.*, 2005 ▸). Image pre-processing [frame alignment with *MotionCor*2 (Zheng *et al.*, 2017 ▸) and CTF estimation using *CTFFIND*4 (Rohou & Grigorieff, 2015 ▸)] were performed using the *Appion* processing environment (Lander *et al.*, 2009 ▸) for real-time feedback during data collection. Images were collected on a Talos Arctica transmission electron microscope (Thermo Fisher) operating at 200 kV with a gun lens of 6, a spot size of 6, a 70 µm C2 aperture and a 100 µm objective aperture using beam-image shift. Movies were collected using a K2 direct electron detector (Gatan) operating in counting mode at 45 000×, corresponding to a physical pixel size of 0.91 Å per pixel, with a 10 s exposure using 200 ms per frame. Using an exposure rate of 4.204 e per pixel per second, each movie had a total dose of approximately 42 e Å^−2^ for the 2111 movies over a defocus of 0.8–2 µm.

### 2.3. Pre-processing   

Movies were aligned using *RELION*-3.0 (3.0-beta-2; Zivanov *et al.*, 2018 ▸) motion correction with five patches in both the *X* and *Y* directions and a *B* factor of −150 Å^2^ without binning. Following motion correction, CTF estimation was performed with *CTFFIND*4 (Rohou & Grigorieff, 2015 ▸) using exhaustive search for a defocus range of 0.5–5.0 µm (0.05 µm step size) and an astigmatism search range of 0.5 µm within a resolution range of 6–30 Å. The combination of a large astigmatism search with exhaustive searches led to many overestimates of CTF resolution fits for this data set. Therefore, in order to remove micrographs automatically, we utilized our recently developed *MicAssess* program (Li *et al.*, 2020 ▸) to remove all empty and bad micrographs. This removed 685 micrographs, leaving 1426 micrographs for particle picking. Particles were picked from aligned micrographs using *crYOLO* (Wagner *et al.*, 2019 ▸) general model PhosaurusNet with an anchor size of 98 × 98 pixels.

### 2.4. Single-particle analysis without aberration correction   

For 2D classification, 718 578 particles were extracted with an unbinned box size of 300 pixels and subsequently binned to 2.73 Å (box size 100 pixels). Particles were then subjected to 2D classification into 100 classes using *RELION*-3.0.2 (*T* = 2; Iter = 25). After selecting particles from the best classes, 275 487 particles underwent 3D classification into five classes using *RELION*-3.0.2 (*T* = 4; Iter = 25) with EMDB entry EMD-8743 (Herzik *et al.*, 2017 ▸) as a reference model. Following the selection of the best classes, 186 841 particles were centered and re-extracted at 0.91 Å per pixel. This stack was used for 3D refinement to obtain a post-processed structure with a resolution of 4.9 Å and a *B* factor of −347 Å^2^.

### 2.5. Aberration correction and particle polishing   

Particles were grouped into optics groups based on beam-image-shift values obtained from the *Leginon* database. In order to group particles into discrete optics groups, the entire file of beam-image-shift values was divided into 5 × 5, 10 × 10 or 20 × 20 groups. The first two beam-tilt estimation steps (CTF refine #1 and #2; Fig. 4) used *RELION*-3.0 (3.0-beta-2). The subsequent steps (Bayesian polishing and CTF refine #3) used *RELION*-3.1 (version 30001). All steps in aberration correction and polishing are described in Fig. 4. Aberration correction and polishing did not improve the resolution further than the final 2.8 Å resolution aldolase structure. We also tested whether using predicted beam tilts from CTF refine #1 could improve the resolution of the final reconstruction; however, this did not improve the data-set resolution (data not shown).

### 2.6. Model building and refinement   

The coordinates of rabbit aldolase (PDB entry 5vy5; Herzik *et al.*, 2017 ▸) were docked into each map using *phenix.dock_in_map* in *Phenix* (Liebschner *et al.*, 2019 ▸). Structure refinement and model validation were performed using *phenix.real_space_refine* (Afonine *et al.*, 2018 ▸). The same docking and refinement parameters were used for each map. To make figures showing map density, *phenix.map_box* was used to restrict the map shown to specific stretches of residues. Root-mean-square deviation (r.m.s.d.) values comparing all atoms between structures were calculated using a least-squares fit in *Coot* (Emsley *et al.*, 2010 ▸). The *PyMOL* Molecular Graphics System (version 2.1; Schrödinger) was used to render images showing these structures and *ChimeraX* was used to render the map images (Goddard *et al.*, 2018 ▸; Pettersen *et al.*, 2020 ▸).

## 3. Results   

### 3.1. Beam-image-shift data collection and analysis   

**Figure 1:**
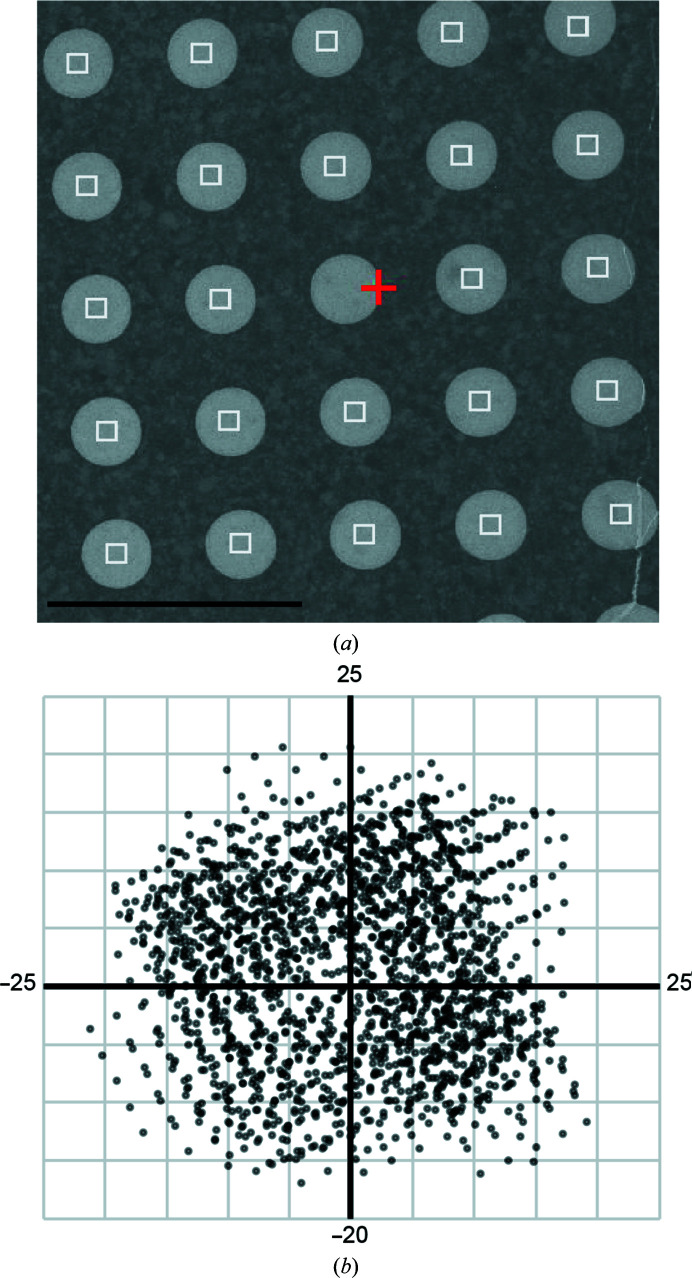
Data-collection strategy for micrographs collected with beam-image shift. (*a*) Representative image at intermediate magnification. Red cross, focus area; white squares, exposures. The scale bar is 5 µm. Each exposure was collected with image-shift beam tilt. (*b*) Overview of image-shift values from *Leginon* for the beam-tilt data set. The units shown are µm.

**Figure 2:**
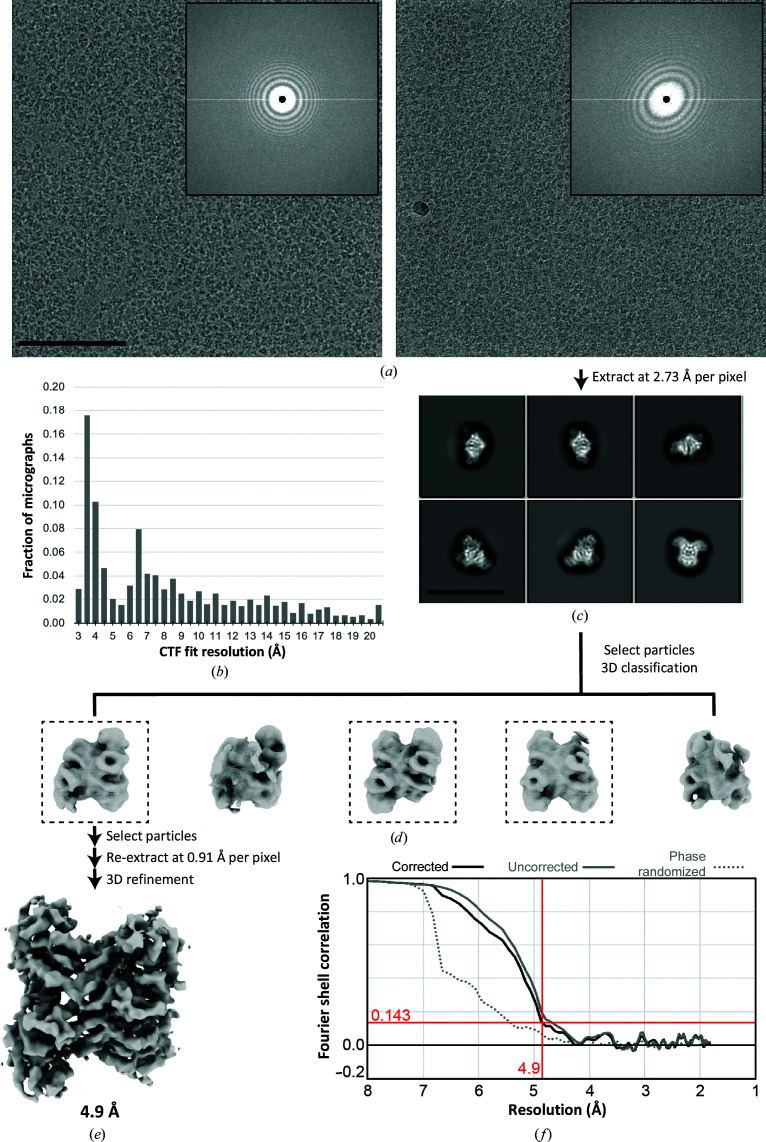
Single-particle analysis of aldolase without beam-tilt correction. (*a*) Representative micrographs with minimal (left) and obvious (left) beam-tilt-induced objective astigmatism. Inset: cropped power spectrum. The scale bar is 100 nm. (*b*) Histogram of CTF resolution limits across the data set using *CTFFIND*4. (*c*) Representative 2D class averages calculated using *RELION*. The scale bar is 200 Å. (*d*) 3D classification results for selected particles after 2D classification. Dashed boxes indicate classes with particles used for subsequent 3D refinement. (*e*) Sharpened reconstruction after 3D refinement using *RELION* filtered to 4.9 Å resolution. (*f*) FSC curves for final reconstruction.

To increase the speed of data collection on the Talos Arctica, we utilized beam-image shift instead of traditional stage movement. While it greatly increases the throughput, we sought to determine how to correct for any aberration from beam-image shift. In order to test the impact of beam-image shift on data quality, we set up the automated data-collection system to target 5 × 5 holes with beam-image shift [Fig. 1 ▸(*a*)]. At medium magnification [Fig. 1 ▸(*a*)], we typically focused on the middle hole, which was followed by beam-image shift with distances up to 5 µm away from the beam center. After collecting 2111 micrographs over 18 h with 10 s exposures, we obtained a large range of beam-image-shift micrographs that provided a near-continuous distribution across the 10 × 10 µm area [Fig. 1 ▸(*b*)]. Interestingly, while many micrographs showed minimal objective astigmatism [Fig. 2 ▸(*a*), left], a large percentage of the data set showed exaggerated objective astigmatism [Fig. 2 ▸(*a*), right] which can be induced by a large amount of axial beam tilt (Glaeser *et al.*, 2011 ▸).

Following data collection, the aldolase beam-image-shift data were analyzed using standard single-particle processing (Fig. 2 ▸). This involved estimating the contrast transfer function (CTF) using *CTFFIND*4 (Rohou & Grigorieff, 2015 ▸), which yielded CTF fits to higher than 4 Å resolution for the majority of the micrographs [Fig. 2 ▸(*b*)]. After picking and extracting particles, 2D classification showed clear secondary-structure features [Fig. 2 ▸(*c*)] consistent with previous work on aldolase (Herzik *et al.*, 2017 ▸; Kim *et al.*, 2018 ▸). After selecting particles from class averages exhibiting high-resolution features, we performed 3D classification in order to obtain a homogenous population of aldolase particles with all four subunits intact [Fig. 2 ▸(*d*)]. Using these selected particle coordinates, particles were re-extracted at the full pixel size (0.91 Å per pixel) and subjected to 3D refinement in *RELION*. The refined structure reached a resolution of only 4.9 Å [Figs. 2 ▸(*e*) and 2 ▸(*f*)], which is significantly less than the published resolution of ∼3 Å (Kim *et al.*, 2018 ▸; Herzik *et al.*, 2017 ▸). This suggested that the aberrations from beam tilt induced by beam-image-shift data collection are likely to limit the resolution of the final structure.

### 3.2. Beam-tilt correction of aldolase cryo-EM micrographs   

**Figure 3:**
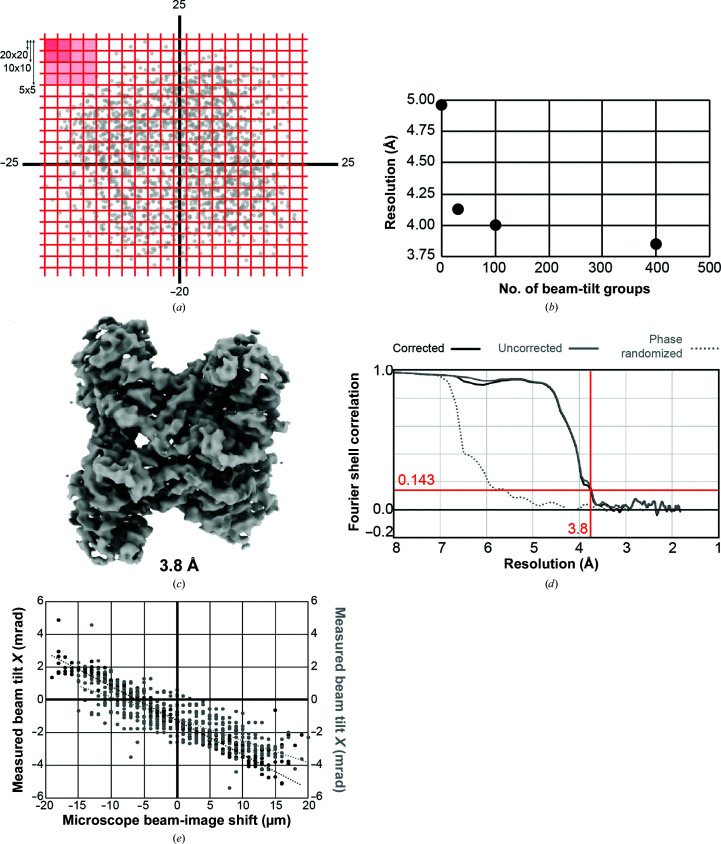
Improved resolution and map quality using beam-tilt refinement. (*a*) Strategy for grouping micrographs. Micrographs were grouped into 25 groups (5 × 5), 100 groups (10 × 10) and 400 groups (20 × 20). (*b*) The effect of group size on beam-tilt refinement and subsequent resolution estimation for refined 3D structures. (*c*) Sharpened 3D reconstruction for particles placed into 400 micrograph groups filtered to 3.8 Å resolution. (*d*) FSC curves for 3D reconstruction in (*c*). (*e*) Beam-tilt measurements for each group displayed with respect to microscope beam-image shift for *X* coordinates (black) and *Y* coordinates (gray). Dashed lines show least-squares fit where *R* ^2^ = 0.96 (beam tilt *X*) and *R* ^2^ = 0.64 (beam tilt *Y*).

After determining a refined 3D structure of aldolase, we wanted to test whether the beam-tilt refinement option in *RELION* 3.0+ is capable of overcoming such a large degree of axial beam tilt. To use this feature of *RELION*, the micrographs must be grouped into beam-tilt groups. Considering the near-continuously changing beam-image-shift data collection for the entire data set [Fig. 1 ▸(*b*)], beam-image-shift values from *Leginon* were used in order to divide the micrographs into groups [Fig. 3 ▸(*a*)]. This involved dividing data into groups of 25 (5 × 5), 100 (10 × 10) and 400 (20 × 20) based on the amount of beam-image shift in *Leginon* (Supplementary Fig. S1). For each grouping, the particles underwent beam-tilt refinement, 3D refinement and sharpening in *RELION* in order to determine the change in the final resolution of the structure. We saw that grouping into 5 × 5, 10 × 10 and 20 × 20 groups gave a significant increase in the final resolution to 4.1, 4.0 and 3.8 Å, respectively [Fig. 3 ▸(*b*)]. This result indicates that the previously determined structure at 4.9 Å resolution was limited in resolution owing to beam-tilt aberrations that could be partially overcome by grouping the data into beam-tilt groups in *RELION*.

For the micrographs divided into 400 groups, the subsequently refined map showed improved density features and had a gold-standard FSC value of 3.8 Å [Figs. 3 ▸(*c*) and 3 ▸(*d*)]. This indicates that beam-tilt refinement improved the resolution of aldolase significantly from 4.9 to 3.8 Å in a single step.

Using the calculated beam-tilt values from *RELION*, we then compared how the beam tilt changed as a function of microscope beam-image shift [Fig. 3 ▸(*e*)]. This comparison reveals a few key features of this data set. Firstly, without any applied beam-image shift at (0, 0), there was a significant amount of beam tilt present: −1.24 mrad (*X*) and −1.14 mrad (*Y*). Secondly, the change in beam tilt based on the change in beam-image shift [the slope in Fig. 3 ▸(*e*)] was different for the *X* versus *Y* direction: −2.1 × 10^5^ µm mrad^−1^ versus −1.35 × 10^5^ µm mrad^−1^, respectively. Finally, this result also shows that a subset of micrographs have a much larger beam tilt than the majority of micrographs, explaining why some micrographs displayed objective astigmatism owing to high beam tilt [Fig. 2 ▸(*c*)].

**Figure 4:**
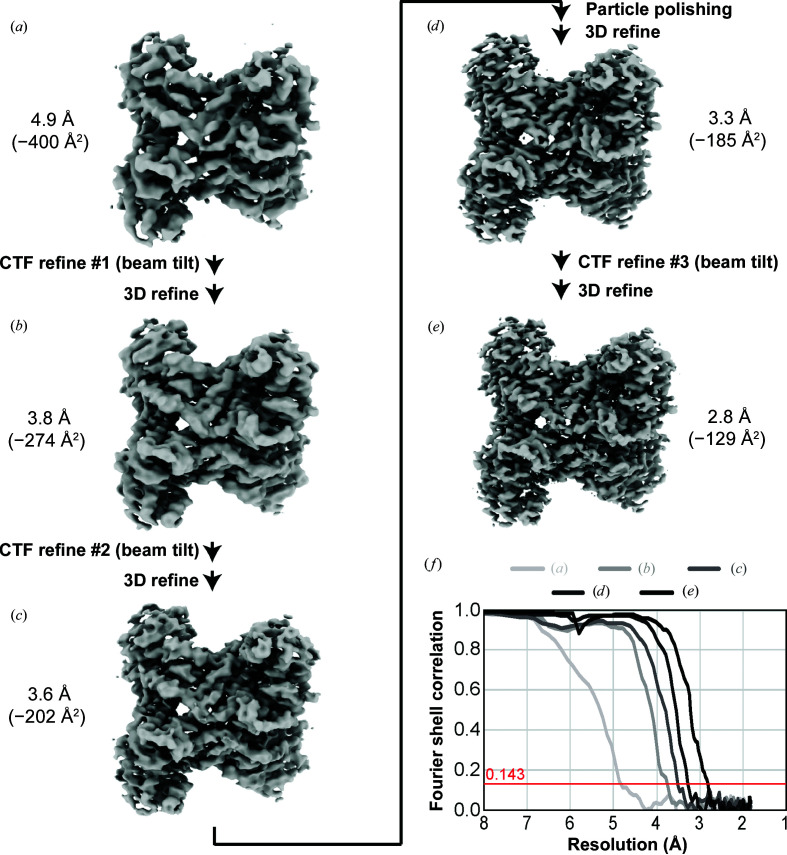
Iterative CTF refinement with particle polishing improves the overall resolution to 2.8 Å. (*a*) Initial 3D structure at 4.9 Å resolution. Following the first CTF refinement and 3D refinement to obtain a structure at 3.8 Å resolution (*b*), continued CTF refinements alongside Bayesian particle polishing allowed resolution and *B*-factor improvements (*c*, *d*, *e*), ultimately allowing the determination of a 2.8 Å resolution structure (*e*). (*f*) FSC curves for 3D reconstructions from (*a*) to (*e*).

Given that the *RELION* beam-tilt estimation step is dependent on the resolution of the 3D reconstruction, we performed iterative beam-tilt refinements and Bayesian particle polishing in order to test whether refinement of beam tilt and particles can further increase the data-set resolution [Fig. 4 ▸, Supplementary Fig. S4(*b*)]. Starting with the 20 × 20 grouped data set at 3.8 Å resolution [Fig. 4 ▸(*b*)], we used this map to recalculate beam tilt for micrographs across the data set. Then, using these new beam-tilt values, we performed another round of 3D refinement. This new structure refined to a higher resolution at 3.6 Å and had a lower *B* factor (−202.0 Å^2^) [Fig. 4 ▸(*c*)], indicating that the per-particle quality has increased. After these two rounds of beam-tilt refinement, we then utilized Bayesian particle polishing in *RELION* (Zivanov *et al.*, 2019 ▸) to further improve the resolution to 3.3 Å (*B* factor −185.1 Å^2^) [Fig. 4 ▸(*d*)]. Then, with these particles, we performed a final beam-tilt calculation, which allowed us to determine a 2.8 Å reconstruction (*B* factor −129.9 Å^2^) [Fig. 4 ▸(*e*)]. This reconstruction could not be improved with further aberration refinements or defocus refinements (data not shown), but our final reconstruction is not limited owing to particles with high defocus [Supplementary Fig. S5(*b*)]. The increase in map quality and model statistics from 4.9 to 2.8 Å resolution [Supplementary Figs. S2 and S4(*b*), Supplementary Table S2] demonstrates that the aberration correction improved the interpretability of the reconstructions. In addition, we compared each of the reconstructions with the atomic model of aldolase (Herzik *et al.*, 2017 ▸) with rigid-body fitting into our electron-density map. The model–map FSC [Supplementary Fig. S4(*a*)] between our maps and the published aldolase model indicates that each iteration of CTF refinement, particle polishing and beam-tilt refinement produces maps that better resemble the existing model.

In order to test whether there were remaining beam-tilt aberrations, we divided the final reconstruction into two subsets: (i) particles with <0.5 mrad measured beam tilt and (ii) particles with >2 mrad measured beam tilt (Supplementary Fig. S3). After matching the number of particles per group to be the same (group 1 had only 20 231 particles), we refined these two groups using *RELION*. Group 1 refined to a higher resolution and a lower *B* factor (3.2 Å and −97 Å^2^, respectively) [Supplementary Fig. S3(*b*)] versus group 2 (3.5 Å and −107 Å^2^, respectively) [Supplementary Fig. S3(*c*)]. This indicates that the data quality for the small measured beam-tilt group is higher than for particles with larger beam tilt.

**Figure 5:**
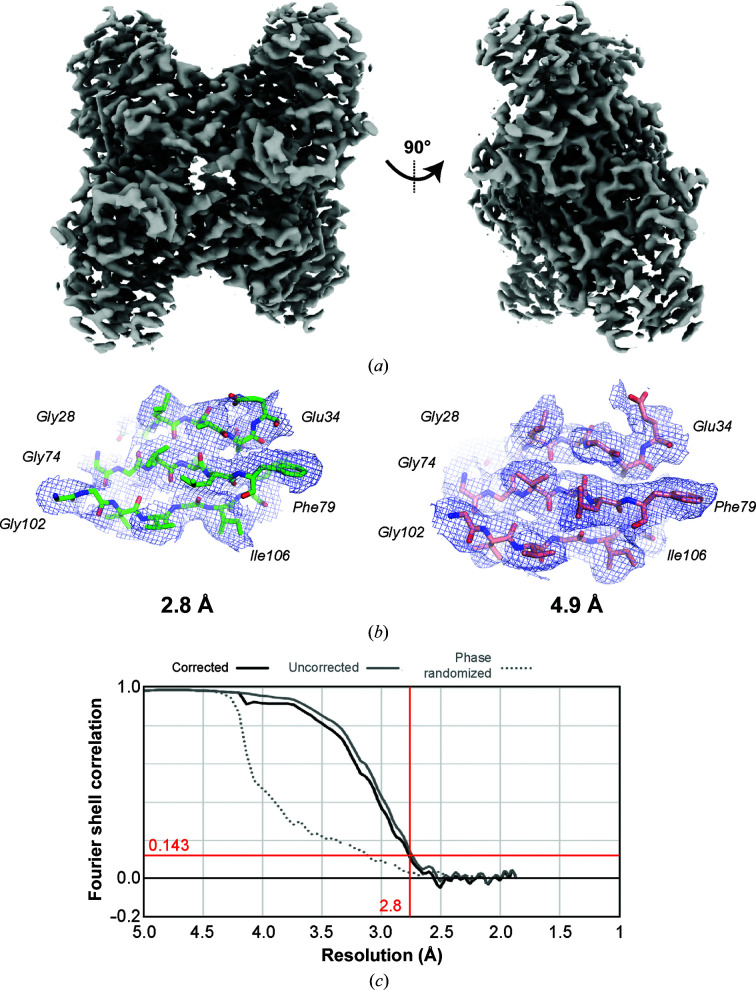
Final aldolase reconstruction at 2.8 Å resolution. (*a*) Sharpened aldolase reconstruction at 2.8 Å resolution. (*b*) Example densities and models for aldolase at 2.8 and 4.9 Å resolution. (*c*) FSC curve for the final reconstruction.

The final structure at 2.8 Å resolution (Fig. 5 ▸) shows dramatically improved density features compared with the original 4.9 Å resolution structure. Specifically, the significantly higher resolution provides unambiguous secondary-structure tracing, whereas the 4.9 Å resolution structure contained many more ambiguities [Fig. 5 ▸(*b*)]. A comparison of model-refinement statistics also highlights the improved map quality for the final 2.8 Å reconstruction (Supplementary Table S2). This structure demonstrates that computational correction of microscope aberrations and particle motion allows sub-3 Å resolution structure determination.

## 4. Discussion   

### 4.1. Single-particle analysis of aldolase with significant microscope aberrations   

The data set analyzed in this work utilized significant beam-image-shift data collection at 200 keV on a Talos Arctica. This strategy introduced significant microscope aberrations into the raw data and was significant enough to cause objective astigmatism in micrographs owing to a large amount of beam tilt [Fig. 2 ▸(*a*), right]. During data collection, we did not perform additional alignments such as those used to set up parallel illumination. We only corrected objective astigmatism and beam-tilt pivot points for automatic focusing. Both of these slight adjustments did not alter the imaging abnormalities, suggesting that the aberrations came from altering the beam-image shift during data collection. In addition, we did not see any significant difference in which micrographs were used in the final reconstructions given that the distribution of image shift per particle did not change significantly during data processing [Supplementary Fig. S5(*a*)].

Despite the presence of significant aberrations, analysis of the resulting aldolase particle stacks allowed 2D and 3D averaging. The 2D class averages obtained from *RELION* for aldolase [Fig. 2 ▸(*c*)] are indistinguishable from previously published aldolase class averages (Herzik *et al.*, 2017 ▸; Kim *et al.*, 2018 ▸), indicating that the aberrations do not affect 7–10 Å resolution class averages. Importantly, however, 3D refinement of the original particle stack does not achieve better than 4.9 Å resolution [Fig. 2 ▸(*e*)], which is much lower than typical aldolase reconstructions, which are within the range 3–4 Å for initial 3D refinements (Herzik *et al.*, 2017 ▸; Kim *et al.*, 2018 ▸). This analysis indicates that microscope aberrations do not affect sample screening and initial 2D averaging; however, the aberrations prevent structure determination at <5 Å.

### 4.2. Significant improvement of resolution through iterative beam-tilt correction   

By taking advantage of microscope aberration correction in *RELION*-3.0+ (Zivanov *et al.*, 2020 ▸), we were able to improve the resolution of aldolase from 4.9 to 2.8 Å. While previous work demonstrated that aberration refinement allows resolution improvements for data at both 300 keV (Zivanov *et al.*, 2018 ▸) and 200 keV (Zivanov *et al.*, 2020 ▸; Wu *et al.*), all data sets analyzed were collected using relatively well aligned instruments. With high-quality starting data, the initial reconstructions prior to aberration correction achieved ∼3 Å resolution (unlike the 4.9 Å resolution in this work). Moreover, the data collected at 200 keV (Wu *et al.*; Herzik *et al.*, 2017 ▸) used stage positioning instead of beam-image shift, further minimizing microscope aberrations in the data set. However, it should be noted that the observed difference in resolution between our work and previous work could have been the result of ice thickness and grid or sample preparation in addition to beam-image-shift corrections.

Using algorithmic improvements in *RELION* (Zivanov *et al.*, 2018 ▸) in combination with Bayesian particle polishing (Zivanov *et al.*, 2019 ▸), we were able to improve the resolution of aldolase to 2.8 Å (Figs. 4 ▸ and 5 ▸). Analysis of the measured beam tilts indicates that there was axial beam tilt present on the instrument prior to using beam-image shift [Fig. 3 ▸(*e*)]. This confirms that the microscope had axial beam tilt prior to data collection; better microscope alignments could have minimized this issue. This said, beam-tilt correction not only fixes off-axis coma but also other coma from imperfect alignment.

Despite utilizing microscope aberration correction and particle polishing, the overall per-particle data quality remained worse than stage position-collected aldolase data. By comparing the final post-processing *B* factor from our data collected using beam-image shift (−52 Å^2^) with aldolase determined from stage positioning (−35 Å^2^; Herzik *et al.*, 2017 ▸), the higher *B* factor for our data indicates that the per-particle signal is lower for our data set. Importantly, for particles with <0.5 mrad beam tilt, we obtained a post-processing *B* factor of −24 Å^2^, indicating that a subset of particles were of comparable or higher quality than the published work (Supplementary Fig. S3). We do not know whether alternative data-processing strategies are needed for beam-image-shift data collection or whether our sample preparation of aldolase is of poorer quality, but further work is needed to verify whether beam-image-shift *B* factors are consistently higher than stage position-collected data at 200 keV.

### 4.3. Data throughput versus data quality   

The main motivation for utilizing beam-image shift for data collection instead of stage positioning is the increased data-collection throughput. For the data set collected here, we were able to obtain a 2.4-fold increase in throughput for beam-image shift when compared with stage positioning: 73 movies per hour (beam-image shift) versus 30 movies per hour (stage positioning). Considering the cost of instrument time, beam-image shift provides 1752 movies per 24 h period versus 720 movies per 24 h period for stage positioning. Indeed, the latest generation of detectors with faster readout stand to triple this throughput for beam-image shift (Alewijnse *et al.*, 2017 ▸; Bromberg *et al.*, 2020 ▸).

Based on our analysis of aldolase, we believe that there is a significant difference between 200 and 300 keV beam-image-shift data collection (for instance where there is not an optical correction on the microscope). At 300 keV, it is possible to use a comparable beam-image shift to that used in this study but instead obtain a structure at ∼3 Å resolution (Zivanov *et al.*, 2018 ▸). For this data set at 300 keV, beam-image shift provides high-resolution structures prior to aberration correction. Unlike this previous study, our aldolase structure collected using beam-image shift at 200 keV was limited in resolution owing to aberrations to 4.9 Å resolution. In order to correct for the aberrations, significant effort was required in order to perform optical grouping and analysis, steps that may be beyond beginner to intermediate *RELION* users.

With these considerations, we advocate beam-image shift at 200 keV for sample screening. This is because we observed high-quality 2D class averages for aldolase despite significant beam tilt, information that is well suited for sample screening (*i.e.* changing buffers, sample concentrations *etc.*). However, this study does indicate that even if a user collected data with significant beam tilt from beam-image-shift data, software-based aberration correction is possible to <3 Å resolution for well behaved samples such as aldolase.

## 5. Data accessibility   

Cryo-EM structures have been deposited in the EMDB with accession codes EMD-22754 [PDB entry 7k9l; Fig. 4 ▸(*e*)], EMD-22755 [PDB entry 7k9x; Fig. 4 ▸(*d*)], EMD-22756 [PDB entry 7ka2; Fig. 4 ▸(*c*)], EMD-22757 [PDB entry 7ka3; Fig. 4 ▸(*b*)] and EMD-22758 [PDB entry 7ka4; Fig. 4 ▸(*a*)]. All movies, micrographs, particle stacks and metadata files have been deposited in EMPIAR as entry EMPIAR-10519.

## Supplementary Material

EMDB reference: aldolase, EMD-22754

EMDB reference: EMD-22755

EMDB reference: EMD-22756

EMDB reference: EMD-22757

EMDB reference: EMD-22758

PDB reference: aldolase, 7k9l

PDB reference: 7k9x

PDB reference: 7ka2

PDB reference: 7ka3

PDB reference: 7ka4

Supplementary Figures and Tables. DOI: 10.1107/S2052252520013482/eh5007sup1.pdf
