# Supplementary material for: High-resolution cryo-EM using beam-image shift at 200 keV
Source: IUCrJ. 2020 Oct 29;7(Pt 6):1179–87. doi: 10.1107/S2052252520013482 (PMC7642776; doi:10.1107/S2052252520013482)
Supplement: Supplementary file 1 [file m-07-01179-sup1.pdf]

IUCrJ

Volume 8 (2021)

Supporting information for article:

High-resolution cryo-EM using beam-image shift at 200 keV

Jennifer N. Cash, Sarah Kearns, Yilai Li and Michael A. Cianfrocco

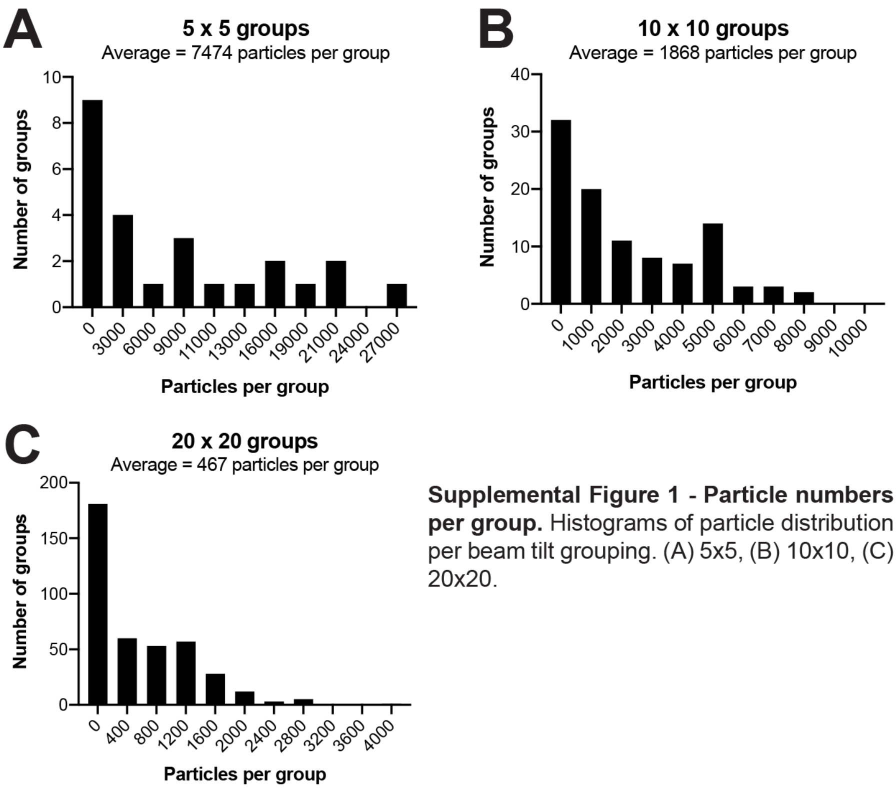

Supplemental Figure 1 - Particle numbers per group. Histograms of particle distribution per beam tilt grouping. (A) 5x5, (B) 10x10, (C) 20x20.

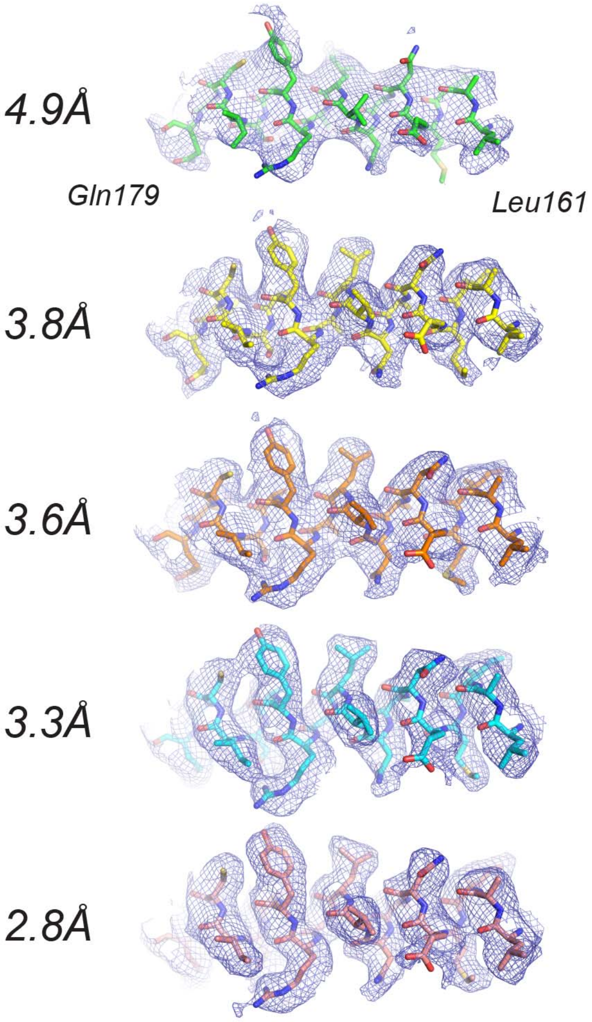

Supplemental Figure 2 - Representative densities from iterative beam-tilt refinements. Sharpened densities with associated models highlight changes in density quality through iterative rounds of beam-tilt refinement corresponding to structures from Figure 4A-4E.

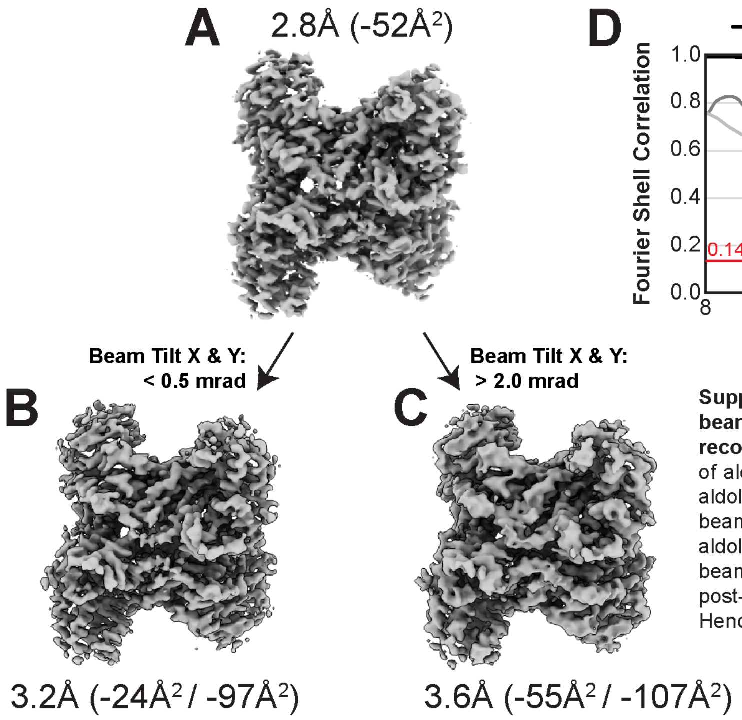

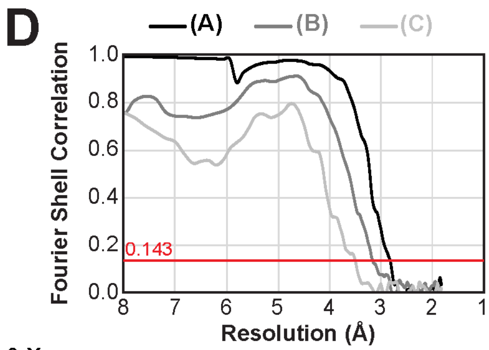

Supplemental figure 3 - Re-analysis of beam tilt subgroups from final reconstruction. (A) Final reconstruction of aldolase at 2.8Å. (B) Reconstruction of aldolase from micrographs with < 0.5 mrad beam tilt at 3.2Å. (C) Reconstruction of aldolase from micrographs with > 2.0 mrad beam tilt at 3.5Å. B-Factors: post-processing B-Factor / Rosenthal-Henderson approach. (D) FSC curves.

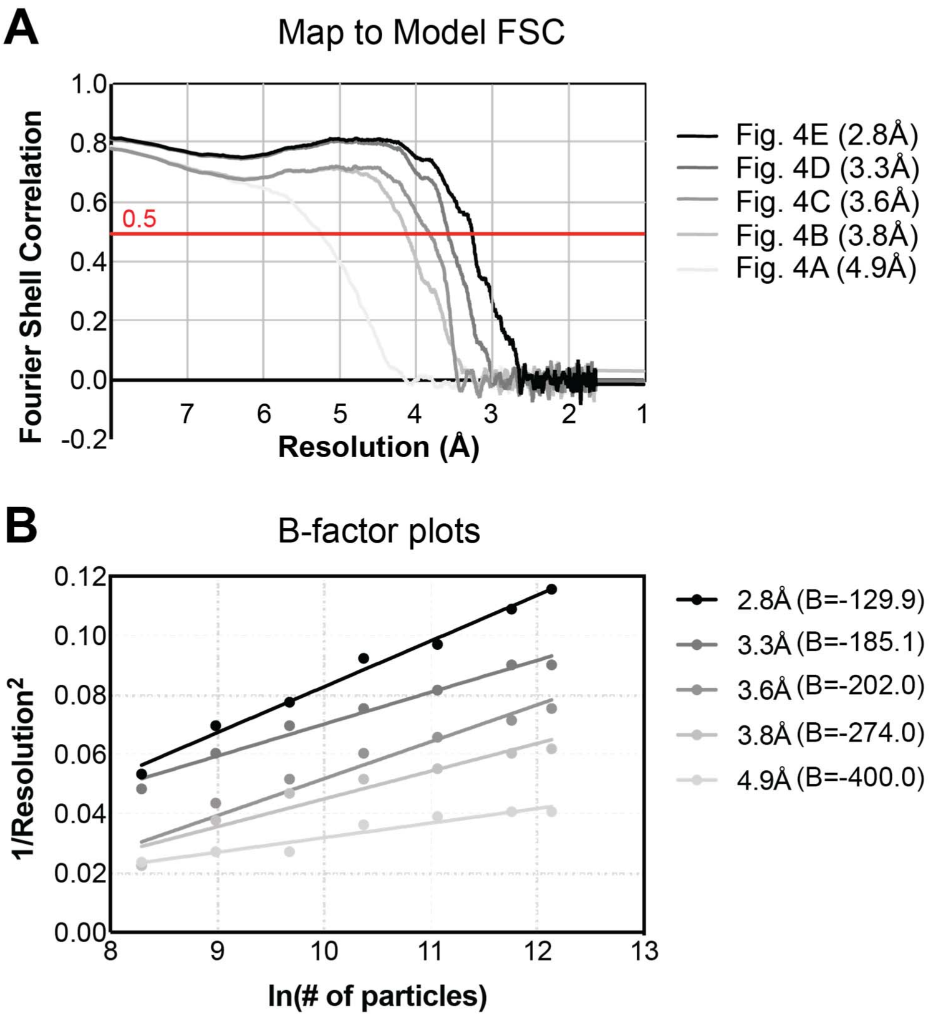

Supplemental figure 4 - Structure validation during data analysis. (A) Map-to-model FSC comparing our maps from Fig. 4A-E with 5vy5 as the model. (B) Rosenthal-Henderson plots showing particle number and resolution to determine B-factors for each reconstruction.

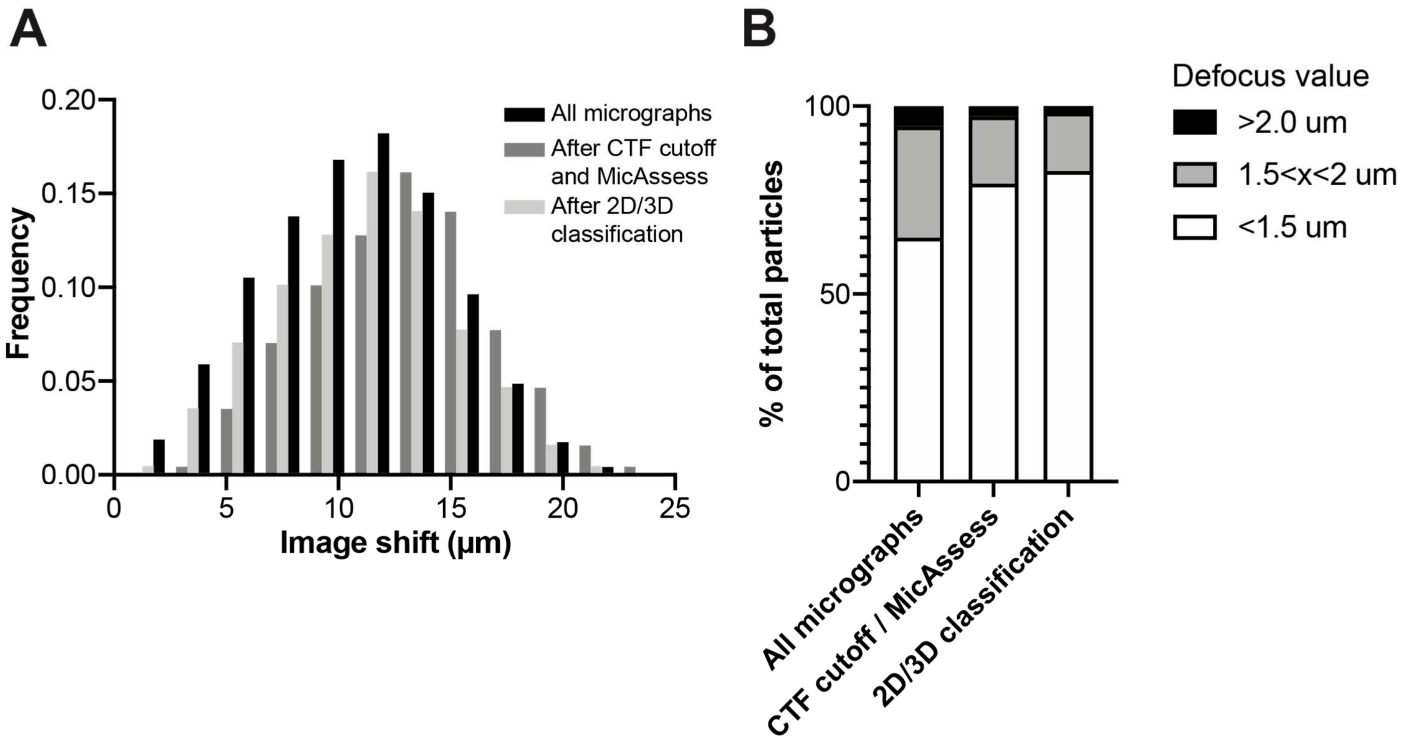

Supplemental figure 5 - Quantification of micrograph image shift and particle defocus. (A) For all micrographs from the dataset (black bars), the distance from (0,0) was determined and plotted as a function of frequency. The same was done for the subset of micrographs kept after CTF cut off and MicAsses (dark grey bars) and 2D/3D classification (light grey bars). (B) For every particle extracted, the defocus was binned into lower than 1.5 um (white), higher than 2 um (black), or inbetween (grey). The same was done for the subset if particles kept after CTFcutoff and MicAssess (500,001) and 2D/3D classification (186,471 particles).

Supplemental Table S1 - Cryo-EM data collection, refinement, and validation statistics.

	**Aldolase (19apr12a)**
**Microscope**	Talos-Arctica
**Detector**	Gatan K2
**Voltage (keV)**	200
**Electron exposure (e$^-$/Å^2)**	43
**Defocus range (µm)**	0.8 - 2
**Data collection mode**	Beam-image shift
**Micrographs collected (per hour)**	73
**Original pixel size (Å)**	0.91
**Symmetry imposed**	D2
**Initial number of micrographs**	2,111
**Final number of micrographs**	569
**Initial particle images (no.)**	718,578
**Final pixel size**	0.91
**Final particle images (no.)**	186,841
**Number of optics groups**	400
**FSC threshold**	0.143
**Final map resolution (Å)**	2.8
**Final post-processing B-Factor (Å^2)**	-52

Supplemental Table S2 - Model building statistics.

**Structure from Figure 4**	**4A**	**4B**	**4C**	**4D**	**4E**
**EMDB/PDB ID**	**22754/7K9L**	**22755/7K9X**	**22756/7KA2**	**22757/7KA3**	**22758/7KA4**
**Resolution (Å)**	4.9	3.8	3.6	3.3	2.8
Postprocessing B-Factor **(Å^2)**	-347	-168	-105	-91	-52
**B-Factor (Å2)** (Rosenthal et. al. 2003)	-400.0	-274.0	-202.0	-185.1	-129.9
**RELION Version**	3.0.2	3.0.8	3.0.8	3.1	3.1
**Bonds (RMSD)**					
Length (Å)	0.007	0.008	0.008	0.004	0.005
Angles (°)	1.197	0.970	0.973	0.767	0.894
**Molprobity score**	2.18	2.21	1.94	1.35	1.86
**Clash score**	9.26	6.36	6.08	3.99	4.23
**Ramachandran plot (%)**					
Outliers	0	0	0	0	0
Allowed	3.52	3.81	4.40	2.93	3.23
Favored	96.48	96.19	95.60	97.07	96.77
**Rotamer outliers (%)**	3.96	6.12	2.52	0.36	3.96
**CaBLAM outliers (%)**	3.54	2.95	2.95	2.95	2.65

**CC (mask)**	0.78	0.81	0.83	0.82	0.82
**CC (box)**	0.67	0.79	0.87	0.76	0.77
**CC (peaks)**	0.58	0.71	0.80	0.72	0.73
**CC (volume)**	0.79	0.81	0.83	0.81	0.81
**RMSD (Å) all atoms compared to Figure 4E**	1.01	0.80	0.84	0.62	-
**Resolution values of map-to-model FSC from Supplemental Figure 4 (Å)**	5.3	4.1	3.8	3.6	3.2
